# Supplementary material for: Multi‐dataset identification of innovative feature genes and molecular mechanisms in keratoconus
Source: J Cell Mol Med. 2024 Sep 19;28(18):e70079. doi: 10.1111/jcmm.70079 (PMC11412914; doi:10.1111/jcmm.70079)
Supplement: Supplementary file 2 — Material S2. [file JCMM-28-e70079-s002.zip › 7.GSEA analysis/3.DBNDD1/KEGG_PROSTATE_CANCER.html]

Details for gene set KEGG\_PROSTATE\_CANCER[GSEA]

|  || Dataset | DBNDD1.group.cls#h\_versus\_l |
| Phenotype | group.cls#h\_versus\_l |
| Upregulated in class | l |
| GeneSet | KEGG\_PROSTATE\_CANCER |
| Enrichment Score (ES) | -0.3389951 |
| Normalized Enrichment Score (NES) | -1.4101024 |
| Nominal p-value | 0.07058824 |
| FDR q-value | 0.6751994 |
| FWER p-Value | 0.887 |
Table: GSEA Results Summary

  

Fig 1: Enrichment plot: KEGG\_PROSTATE\_CANCER      
 Profile of the Running ES Score & Positions of GeneSet Members on the Rank Ordered List

  

| SYMBOL | TITLE | RANK IN GENE LIST | RANK METRIC SCORE | RUNNING ES | CORE ENRICHMENT || 1 | PIK3R5 | na | 35 | 0.708 | 0.0701 | No |
| 2 | INSRR | na | 1735 | 0.318 | 0.0530 | No |
| 3 | PDGFRA | na | 3213 | 0.243 | 0.0348 | No |
| 4 | CCNE2 | na | 4812 | 0.186 | 0.0073 | No |
| 5 | AR | na | 6232 | 0.144 | -0.0191 | No |
| 6 | INS | na | 6241 | 0.144 | -0.0049 | No |
| 7 | E2F2 | na | 6645 | 0.133 | -0.0031 | No |
| 8 | PIK3R1 | na | 6912 | 0.126 | 0.0019 | No |
| 9 | IGF1R | na | 8164 | 0.100 | -0.0242 | No |
| 10 | SOS1 | na | 8586 | 0.093 | -0.0270 | No |
| 11 | CREBBP | na | 9242 | 0.082 | -0.0377 | No |
| 12 | PDGFA | na | 9340 | 0.081 | -0.0324 | No |
| 13 | AKT2 | na | 9388 | 0.080 | -0.0257 | No |
| 14 | CCND1 | na | 9676 | 0.076 | -0.0263 | No |
| 15 | MDM2 | na | 9756 | 0.075 | -0.0211 | No |
| 16 | MAPK3 | na | 9865 | 0.073 | -0.0169 | No |
| 17 | PDGFRB | na | 10046 | 0.071 | -0.0150 | No |
| 18 | RB1 | na | 10147 | 0.069 | -0.0109 | No |
| 19 | IGF1 | na | 10555 | 0.064 | -0.0162 | No |
| 20 | MAP2K2 | na | 10931 | 0.059 | -0.0211 | No |
| 21 | PTEN | na | 10959 | 0.059 | -0.0160 | No |
| 22 | PDPK1 | na | 11670 | 0.050 | -0.0315 | No |
| 23 | PIK3R2 | na | 11699 | 0.050 | -0.0272 | No |
| 24 | CREB3 | na | 12277 | 0.045 | -0.0394 | No |
| 25 | MTOR | na | 13075 | 0.038 | -0.0586 | No |
| 26 | ERBB2 | na | 13132 | 0.037 | -0.0565 | No |
| 27 | SOS2 | na | 13580 | 0.033 | -0.0661 | No |
| 28 | TP53 | na | 13962 | 0.030 | -0.0740 | No |
| 29 | KRAS | na | 14127 | 0.029 | -0.0758 | No |
| 30 | PIK3R3 | na | 14144 | 0.029 | -0.0734 | No |
| 31 | HSP90AA1 | na | 14230 | 0.028 | -0.0730 | No |
| 32 | BRAF | na | 15471 | 0.019 | -0.1069 | No |
| 33 | FOXO1 | na | 15621 | 0.018 | -0.1093 | No |
| 34 | CREB3L1 | na | 15797 | 0.017 | -0.1126 | No |
| 35 | CTNNB1 | na | 16378 | 0.013 | -0.1280 | No |
| 36 | PIK3CA | na | 17073 | 0.009 | -0.1472 | No |
| 37 | CCNE1 | na | 17317 | 0.007 | -0.1535 | No |
| 38 | GSTP1 | na | 17472 | 0.006 | -0.1572 | No |
| 39 | PIK3CB | na | 18338 | 0.000 | -0.1822 | No |
| 40 | CHUK | na | 18953 | -0.004 | -0.1995 | No |
| 41 | HRAS | na | 18984 | -0.004 | -0.1999 | No |
| 42 | RAF1 | na | 19005 | -0.005 | -0.2000 | No |
| 43 | AKT1 | na | 19066 | -0.005 | -0.2013 | No |
| 44 | EGF | na | 19333 | -0.007 | -0.2082 | No |
| 45 | CASP9 | na | 19881 | -0.012 | -0.2228 | No |
| 46 | ATF4 | na | 20029 | -0.013 | -0.2257 | No |
| 47 | TCF7L2 | na | 20073 | -0.014 | -0.2256 | No |
| 48 | BAD | na | 20453 | -0.017 | -0.2348 | No |
| 49 | CREB3L3 | na | 20550 | -0.018 | -0.2358 | No |
| 50 | EGFR | na | 22041 | -0.033 | -0.2756 | No |
| 51 | GRB2 | na | 22294 | -0.035 | -0.2793 | No |
| 52 | IKBKB | na | 22300 | -0.035 | -0.2759 | No |
| 53 | GSK3B | na | 22452 | -0.037 | -0.2766 | No |
| 54 | CREB3L4 | na | 22477 | -0.037 | -0.2736 | No |
| 55 | MAPK1 | na | 22705 | -0.040 | -0.2762 | No |
| 56 | TGFA | na | 22844 | -0.041 | -0.2760 | No |
| 57 | HSP90AB1 | na | 22914 | -0.042 | -0.2738 | No |
| 58 | CDKN1B | na | 22949 | -0.042 | -0.2705 | No |
| 59 | IKBKG | na | 23063 | -0.044 | -0.2694 | No |
| 60 | TCF7 | na | 23571 | -0.051 | -0.2789 | No |
| 61 | CREB3L2 | na | 23589 | -0.051 | -0.2743 | No |
| 62 | E2F3 | na | 24031 | -0.057 | -0.2813 | No |
| 63 | EP300 | na | 24292 | -0.060 | -0.2828 | No |
| 64 | CREB1 | na | 24296 | -0.060 | -0.2768 | No |
| 65 | ARAF | na | 24817 | -0.068 | -0.2850 | No |
| 66 | PIK3CD | na | 24971 | -0.070 | -0.2823 | No |
| 67 | FGFR2 | na | 25276 | -0.075 | -0.2836 | No |
| 68 | NFKB1 | na | 25842 | -0.084 | -0.2914 | No |
| 69 | HSP90B1 | na | 26345 | -0.095 | -0.2964 | No |
| 70 | TCF7L1 | na | 26813 | -0.104 | -0.2995 | No |
| 71 | NRAS | na | 27014 | -0.108 | -0.2944 | No |
| 72 | PIK3CG | na | 27455 | -0.118 | -0.2952 | No |
| 73 | NFKBIA | na | 27480 | -0.118 | -0.2840 | No |
| 74 | E2F1 | na | 27800 | -0.126 | -0.2806 | No |
| 75 | CDK2 | na | 28335 | -0.139 | -0.2820 | No |
| 76 | RELA | na | 28616 | -0.147 | -0.2754 | No |
| 77 | MAP2K1 | na | 29246 | -0.164 | -0.2770 | No |
| 78 | KLK3 | na | 31394 | -0.236 | -0.3152 | Yes |
| 79 | BCL2 | na | 31549 | -0.242 | -0.2954 | Yes |
| 80 | CDKN1A | na | 31724 | -0.250 | -0.2753 | Yes |
| 81 | PDGFD | na | 32416 | -0.288 | -0.2663 | Yes |
| 82 | LEF1 | na | 32857 | -0.317 | -0.2471 | Yes |
| 83 | SRD5A2 | na | 32869 | -0.319 | -0.2154 | Yes |
| 84 | AKT3 | na | 33656 | -0.385 | -0.1994 | Yes |
| 85 | FGFR1 | na | 33845 | -0.409 | -0.1637 | Yes |
| 86 | NKX3-1 | na | 33856 | -0.411 | -0.1227 | Yes |
| 87 | PDGFB | na | 34011 | -0.431 | -0.0838 | Yes |
| 88 | PDGFC | na | 34154 | -0.452 | -0.0425 | Yes |
| 89 | CREB5 | na | 34560 | -0.587 | 0.0048 | Yes |
Table: GSEA details [plain text format]

  

Fig 2: KEGG\_PROSTATE\_CANCER      
 Blue-Pink O' Gram in the Space of the Analyzed GeneSet

  

Fig 3: KEGG\_PROSTATE\_CANCER: Random ES distribution      
 Gene set null distribution of ES for **KEGG\_PROSTATE\_CANCER**
